# Inactivation of Shiga Toxin-Producing *Escherichia coli* in Refrigerated and Frozen Meatballs Using High Pressure Processing

**DOI:** 10.3390/microorganisms8030360

**Published:** 2020-03-03

**Authors:** Anna C. S. Porto-Fett, Armitra Jackson-Davis, Lamin S. Kassama, Marciauna Daniel, Michelle Oliver, YangJin Jung, John B. Luchansky

**Affiliations:** 1 United States Department of Agriculture, Agricultural Research Service, Eastern Regional Research Center, 600 East Mermaid Lane, Wyndmoor, PA 19038, USA; yangjin.jung@usda.gov (Y.J.); john.luchansky@usda.gov (J.B.L.); 2 Department of Food and Animal Sciences, Alabama Agricultural and Mechanical University, 4900 Meridian St. N, Normal, AL 35762, USA; armitra.davis@aamu.edu (A.J.-D.); lamin.kassama@aamu.edu (L.S.K.); marciauna0420@yahoo.com (M.D.); moliver9@bulldogs.aamu.edu (M.O.)

**Keywords:** Shiga toxin-producing *Escherichia coli* (STEC), high pressure processing, ground meat, meatballs, veal, beef

## Abstract

High pressure processing (HPP) was evaluated to inactivate Shiga toxin-producing *Escherichia coli* (STEC) in raw meatballs. Ground meat (>90% lean) was inoculated (ca. 7.0 log CFU/g) with a rifampicin-resistant cocktail of eight STEC strains (O26:H11, O45:H2, O103:H2, O104:H4, O111:H-, O121:H19, O145:NM, and O157:H7). Inoculated ground beef, ground veal, or a mixture of ground beef, pork, and veal were separately mixed with liquid whole eggs and seasonings, shaped by hand into meatballs (40 g each), and stored at −20 or at 4 °C for at least 18 h. Samples were then exposed to 400 or 600 MPa for 0 to 18 min. There were no differences (*p* > 0.05) in pathogen reduction related to the species of meat used or for meatballs that were refrigerated (0.9 to 2.9 log CFU/g) compared to otherwise similar meatballs that were stored frozen (1.0 to 3.0 log CFU/g) prior to HPP treatment. However, less time was needed to achieve a ≥ 2.0 log CFU/g reduction at 600 MPa (1 to 3 min) compared to 400 MPa (at least 9 min). This work provides new and practically useful information on the use of HPP to inactivate STEC in raw meatballs.

## 1. Introduction

Consumption of under-processed and improperly handled or stored food products contaminated with *Escherichia coli* O157:H7 or non-O157:H7 Shiga toxin-producing cells of *E. coli* (STEC) are responsible for ca. 265,000 illnesses annually (ca. 5300 laboratory confirmed), as well as ca. 3600 hospitalizations and ca. 30 deaths each year [1]. Cells of serotype O157:H7 STEC have been recognized as a human pathogen since the early 1980s; however, cells of a handful of non-O157 STEC serogroups—namely O26, O45, O103, O111, O121, and O145 (also known as “the big six”)—have only more recently been implicated in foodborne illness [2] and are the etiologic agent for 70 to 83% of confirmed non-O157:H7 illness attributed to STEC in the U.S. [1]. As such, the Food Safety and Inspection Service (FSIS) of the U.S. Department of Agriculture (USDA) issued regulations stipulating that cells of serotype O157:H7 and cells of the big six non-O157 STEC serogroups are considered adulterants in non-intact beef and veal products, including ground beef and related products containing comminuted beef such as raw meatballs [3]. For these reasons, much effort has focused on developing and validating interventions to lower the occurrence and levels of cells of the seven regulated STEC serovars in food, and particularly in beef.

STEC illnesses are associated with undercooked or improperly handled not-ready-to-eat (NRTE) and non-intact beef products, as well as with other food commodities such as leafy greens [4]. Regarding physical interventions other than heat for reducing the risk of foodborne illness due to STEC in raw beef, high-pressure processing (HPP; 100 to 1000 MPa for typically <5 min) is used commercially to inactivate microorganisms (reductions of up to five to six log) directly in/on foods without significant degradation of the food components and while preserving the sensory and nutritional qualities of treated foods [5]. As one example, Cutter et al. [6] inoculated irradiated ground beef patties (80:20 or 93:7 (lean:fat)) with 6.0 log CFU/g of a cocktail of serogroup O26, O45, O103, O111, O121, O145, and O157:H7 cells of STEC and stored these patties at 4 °C prior to HPP treatment. After four 400 MPa cycles of 1 min each at 17 °C, reductions of ca. three to four log were achieved [6]. As another example, Morales et al. [7] demonstrated that HPP reduced *E. coli* O157:H7 levels in ground beef by 0.82 and 4.39 log CFU/g after 1 and 20 min exposures at 400 MPa, respectively; however, significant changes in color and texture were observed after 10 min of HPP treatment. Nonetheless, HPP is gaining favor and increasing use for enhancing safety and maintaining the wholesomeness of raw and ready-to-eat (RTE) red meat and poultry products [5].

Over the past 20 years, numerous recalls and several illnesses have been linked to improperly cooked ground beef and, to a much lesser extent, ground veal [8,9,10,11]. There has also been a recent outbreak and two recalls linked to meatballs [12,13,14]. The associated outbreak from one of these recalls was responsible for six hospitalizations, 18 illnesses, and one death caused by cells of a serogroup O26 STEC strain [13,14], whereas cells of a serogroup O157:H7 STEC strain were recovered from meatballs associated with the second abovementioned recall [12]. The reasons for these recalls of meatballs ranged from “extraneous material”, “undetermined substances/allergens”, and “without the benefit of federal inspection” to the presence of a pathogen such as *Listeria monocytogenes*, *Salmonella*, or STEC [12,15,16,17]. As such, if meatballs harbored STEC, albeit it at low frequency and at low levels, and if such meatballs were not properly cooked, handled, and stored, then such products could cause human illness. Although studies were published on the effect of HPP on inactivation of serotype O157:H7 strains of *E. coli* in ground meats [18,19], far less has been published on HPP inactivation of the big six non-O157 STEC serogroups in raw red meat and poultry products. Data are especially lacking for application of HPP to enhance the safety and quality of multi-species and multi-ingredient raw meats containing beef such as meatballs. Therefore, we evaluated the effect of different pressure levels and treatment times for HPP on inactivation of STEC in raw meatballs prepared with veal, beef, or a veal-beef-pork mixture.

## 2. Materials and Methods

### 2.1. Preparation of Bacterial Strains

The following eight rifampin-resistant (100 µg/mL; Sigma Chemical Company, St. Louis, MO, USA) strains of Shiga toxin-producing *Escherichia coli* (STEC-8) used to inoculate raw meat were prepared and maintained as described by Porto-Fett et al. [20]: (i) H30 (serotype O26:H11), (ii) JBI-95 (serotype O111:H-), (iii) CDC 96-3285 (serotype O45:H2), (iv) CDC 90-3128 (serotype O103:H2), (v) ATCC BAA-2326 (serotype O104:H4), (vi) CDC 97-3068 (serotype O121:H19), (vii) 83-75 (serotype O145:NM), and (viii) USDA-FSIS 011-82 (serotype O157:H7).

### 2.2. Preparation and Inoculation of Meatballs

Meatballs were prepared and inoculated as previously described [20]. Briefly, freshly processed and finely ground veal (veal; ca. 97:3% (lean:fat)); finely ground beef (beef; ca. 90:10% (lean:fat)); or finely ground beef, veal, and pork (meat mix containing ca. 1/3 of each species of meat; ca. 90:10% (lean:fat)) were separately inoculated with the rifampicin-resistant STEC-8 cocktail (1 mL of inoculum to 100 g of ground meat) to achieve an initial inoculum of ca. 7.0 log CFU/g. The inoculated ground veal, ground beef, or ground meat mix (ca. 4.5 kg each) were separately combined with pasteurized liquid whole eggs (900 mL; EggBeaters^®^; ConAgra Foods Inc., Omaha, NE, USA) and with flavored bread crumbs (850 g; Cento^®^; Cento Fine Foods Inc., Thorofare, NJ, USA) using a commercial mixer (Univex SRM12; Univex, Salem, NH, USA). Portions (40 g each) of the inoculated batter were shaped into balls by hand, placed individually into nylon-polyethylene bags (Koch Supplies, Kansas City, MO, USA) and vacuum-sealed to 950 mBar. Each bag was then placed into a second vacuum-sealed nylon-polyethylene bag and stored either at –20 °C (i.e., frozen) or at 4 °C (i.e., refrigerated) for 16 to 18 h, before being subjected to pressurization as described below [21].

### 2.3. High Pressure Processing Treatments

Meatballs inoculated with the STEC-8 cocktail were subjected to 400 MPa (58,015 psi) for 0, 3, 6, 9, or 12 min (fresh meatballs) or 0, 9, 12, 15, or 18 min (frozen meatballs) or 600 MPa (87,023 psi) for 0, 0.5, 1, 1.5, or 3 min (fresh meatballs) or 0, 1.5, 3, 6, or 9 min (frozen meatballs) in a 2 L capacity HPP unit (Model 2 L; Avure Technologies, Kent, WA, USA) essentially as described [21]. The pressure release times were instantaneous. The average initial temperature of the water in the pressure vessel was 19.2 °C (±2.2 °C) and the temperature range attained after pressurizing to 400 or 600 MPa was 22.4 to 27.6 °C or 27.6 to 32.4 °C, respectively. After pressurization, meatball samples were removed from the HPP chamber and immediately placed on ice until microbiological analyses were conducted (within ≤ 30 min).

### 2.4. Microbiological Analyses

After HPP treatment, cells of STEC were recovered from pressure-treated meatballs by aseptically transferring each sample into a filter bag (Type XX-C003; Microbiology International, Frederick, MD, USA) containing 60 mL of sterile 0.1% peptone water (Difco, Becton, Dickinson Co., Sparks, MD, USA) and macerating for 2 min at 230 rpm in a stomacher (Stomacher 400; Seward, Cincinnati, OH, USA). Appropriate serial dilutions of the filtrate were prepared using sterile 0.1% peptone water before 0.1 mL aliquots were surface plated in duplicate onto sorbitol-MacConkey (SMAC; Difco) agar plates plus rifampicin (100 µg/mL). Plates were incubated for 24 h at 37 °C before surviving cells typical for STEC were enumerated. When testing negative for the pathogen by direct plating (≤ 0.40 log CFU/g), samples were enriched as described previously [22].

### 2.5. Statistical Analyses

The SAS system (Version 9.3; SAS Institute, Cary, NC, USA) was used to determine statistically significant differences among pressure times (i.e., 0 to 18 min), pressure levels (i.e., 400 and 600 MPa), state of meat (i.e., refrigerated and frozen), and type of meat (i.e., veal, beef, or multi-species mix). At each of the two pressure levels tested, means and standard deviations were calculated from individual sets of data for each of the three separate trials using triplicate samples at each time interval. Analysis of variance (ANOVA) was used to determine the effects and interactions of the factors on the log reduction values. Differences in lethality observed for each time, pressure level, type of meat, state of meat, and/or combinations thereof were tested for significance at *p* < 0.05 using the Sidak test. The D-values represent the absolute value of the inverse of the linear inactivation rate of the surviving cell fraction.

## 3. Results

In general, regardless of the type of meat used in the formulation (i.e., veal only, beef only, or a mix of veal, beef, and pork), the state of the meat (i.e., refrigerated vs. frozen) just prior to treatment via HPP, or the level of pressure applied (i.e., 400 vs. 600 MPa), longer treatment times produced a greater reduction in the levels of STEC-8. More specifically, when meatballs were pressurized at 400 MPa, there was no significant (*p* > 0.05) difference in the inactivation of STEC-8 between the state of meat or between the types of meat/formulations used to prepare refrigerated and frozen meatballs. However, the variables of time and time × state of the meat had a significant effect (*p* < 0.05) on lethality of STEC when meatballs were pressurized at 400 MPa for up to 18 min. Likewise, with the exception of the type of meat (*p* > 0.05) used to prepare the meatballs, we observed a significant (*p* < 0.05) difference between the state of meat, pressurization times, or the effect of time × state of the meat on the inactivation of STEC-8 when meatballs were subjected to 600 MPa.

For refrigerated meatballs pressurized at 400 MPa, statistical differences (*p* < 0.05) for inactivation of STEC-8 were observed within 3 min when compared with otherwise similar meatballs pressurized for 6, 9, or 12 min, but not (*p* > 0.05) between meatballs that were pressurized for 6 and 9 min or that were pressurized for 9 and 12 min. Likewise, statistical differences (*p* < 0.05) in thermal inactivation of STEC-8 were observed for frozen meatballs that were treated for 18 min at 400 MPa compared with otherwise similar meatballs pressurized for 3 or 9 min, but not for 15 min. For refrigerated meatballs pressurized at 600 MPa, inactivation of STEC-8 was similar (*p* > 0.05) in meatballs treated for 0.5 min when compared to 1 min, but significantly (*p* < 0.05) less when compared to the inactivation observed in meatballs treated for 1.5 or 3 min. For frozen meatballs, statistical differences (*p* < 0.05) in thermal inactivation of STEC-8 were observed among all pressurization times tested using 600 MPa. The pH and the water activity (a_w_) of the untreated meatballs ranged from 5.19 to 5.92 and 0.980 to 0.984, respectively (data not shown).

**Figure 1:**
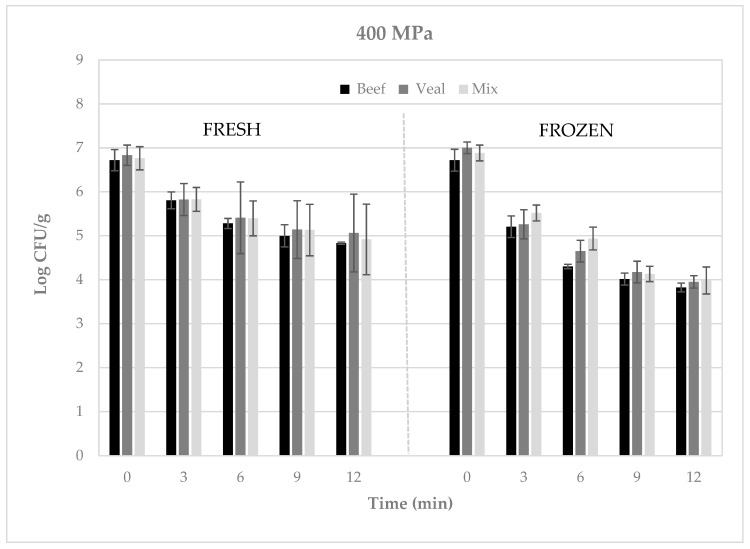
Recovery of Shiga toxin-producing *Escherichia coli* (log CFU/g) from refrigerated or frozen raw meatballs following pressure treatment at 400 MPa. Error bars represent the standard deviation of the mean (N = 3, *n* = 3).

**Figure 2:**
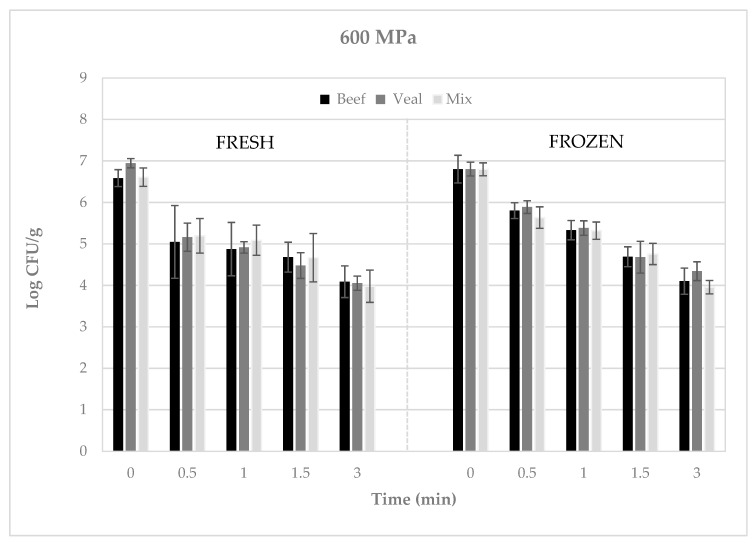
Recovery of Shiga toxin-producing *Escherichia coli* (log CFU/g) from refrigerated or frozen raw meatballs following pressure treatment at 600 MPa. Error bars represent the standard deviation of the mean (N = 3, *n* = 3).

Regardless of the type of meat, when refrigerated meatballs were subjected to 400 MPa for 3 to 12 min, pathogen numbers decreased by ca. 0.9 to 1.9 log CFU/g (Figure 1), whereas when subjected to 600 MPa for 0.5 to 3 min, reductions of ca. 1.4 to 2.9 log CFU/g were achieved (Figure 2). Likewise, when frozen meatballs were pressurized at 400 MPa for 9 to 18 min, pathogen numbers decreased by 1.4 to 3.0 log CFU/g (Figure 1), whereas when subjected to 600 MPa for 1.5 to 9 min, reductions of 0.9 to 2.8 log CFU/g were observed (Figure 2).

The average D-values (Table 1) of STEC in refrigerated meatballs made with veal, beef, or the multi-species mix that were treated at 400 or 600 MPa ranged from 6.54 to 7.10 or 1.24 to 1.45 min, respectively. For frozen veal, beef, or the multi-specie meatballs pressurized at 400 and 600 MPa, the average D-values ranged from 5.69 to 5.90 and 3.56 to 3.80 min, respectively. Our results also established that a 5-log reduction was achieved by pressurizing refrigerated or frozen meatballs at 400 MPa for 32.7 to 35.5 and 28.45 to 29.45 min, respectively, whereas a 5 log reduction was achieved by pressure treating refrigerated or frozen meatballs at 600 MPa for 6.20 to 7.25 or 17.80 to 19.3 min, respectively (data not shown).

## 4. Discussion

Several recalls and illnesses have been linked to ground/non-intact beef and veal. There have also been reports on the recovery of STEC from ground/non-intact beef and veal, with the prevalence being somewhat higher for cells of the big six STEC than serotype O157:H7 STEC on veal than on beef [23]. In addition to a relatively low prevalence, for the infrequent occasions when STEC are recovered from raw beef products, pathogen levels are typically <1.0 log [24]. For these reasons, several investigators have elaborated cooking times and temperatures to appreciably reduce the levels of STEC in ground/non-intact beef and veal [25,26,27], including for thermal inactivation of STEC in meatballs [20]. Regarding the latter, reductions of 0.7 to ≥6.0 log were achieved in 5.5 to 9.0 min when deep frying or oven cooking refrigerated meatballs, whereas 7.5 to 20 min were needed to achieve equivalent reductions for otherwise similar, but frozen, meatballs [20]. Note that meatballs may be purchased/stored as “frozen fully cooked”, “frozen partially cooked”, and/or “frozen raw”. The growth temperature for STEC in foods can range from 7 to 46 °C, but strains of some STEC can survive at refrigeration and freezing temperatures for relatively long periods [28]. Thus, if meatballs are contaminated with STEC, the pathogen may survive during extended frozen storage. It is also quite common for consumers (22%; 44 of 119 volunteer respondents) [29] and non-chain-style restaurants (26%; 66 of 256) [30] to cook raw beef starting from a frozen state. To further lower the risk of STEC-related illness from consumption of under-processed and/or raw meatballs, we evaluated HPP to lower pathogen presence and load. As a clean label intervention, HPP provides producers and processors with an effective non-thermal option to ensure safety, enhance quality, and extend the shelf life of red meat and poultry products.

To the best of our knowledge, there is no research published on the effect of HPP to inactivate STEC in (multi-species) meatballs. Our results, however, are in general agreement with previous studies on HPP inactivation of STEC in ground beef and ground beef patties. For example, Jiang et al. [19] observed a ca. 2.3- to 4.3-log CFU/g reduction of non-O157:H7 and O157:H7 STEC cells in ground beef (80:20% and 90:10% (lean:fat)) samples that were pressurized at 400 MPa during four consecutive cycles (60 s per cycle) at 17 °C. In another study, Hsu et al. [18] reported that reductions of 3.5 to 6.9 log CFU/g were achieved for non-O157:H7 and O157:H7 STEC when ground beef (83:17% (lean:fat)) was subjected to 450 MPa for 5 or 15 min. Black et al. [31] reported that HPP delivered a 3.0-log reduction in STEC O157:H7 in ground beef pressurized at 400 MPa for 10 min at 20 °C. Similarly, Morales et al. [7] quantified reductions of ca. 2.5 log CFU/g of STEC O157:H7 when ground beef was pressurized at 400 MPa in a single cycle for 20 min at 12 °C.

Cells of the seven serogroup-specific STEC regulated by USDA FSIS may be present in ground/non-intact beef and veal. Analysis of raw ground beef components (RGBC) samples, including veal, collected in federal plants in 2018 by USDA FSIS revealed that serotype O157:H7 cells were recovered at a frequency of 0.16% (6 of 3707 samples) from ground beef and at 0.0% (0 of 48 samples) for ground veal [32]. For non-O157 STEC, the pathogen was recovered at a frequency of 0.22% (8 of 3613 samples) for ground beef and at 6.67% (3 of 45 samples) for ground veal [32]. Thus, it is essential to validate interventions to deliver a ≥5.0 log reduction in STEC levels prior to human consumption to decrease the risk of illness associated with undercooked ground/non-intact raw meats such as meatballs.

Although HPP is an effective technology to inactivate foodborne pathogens in meats [33], at times pressurization can adversely affect the quality of the product, and particularly the quality of raw meats. The composition and texture of the raw meat immediately prior to pressurization can also influence the rate of temperature change and the overall transfer of heat, which, in turn, can appreciably impact the tolerance of STEC to heat or pressure and the quality of the product. For example, Gola et al. [34] reported a ≥5.0 log reduction of a cocktail of eight strains of *E. coli* O157:H7 in raw ground beef treated at 700 MPa for 5 min; however, according to these authors, this combination of pressure and time had an unintended negative impact on the color and texture of the product. By empirical observation, pressurization either at 400 or 600 MPa did not have any significant detrimental effects on either the quality or sensory attributes of the product in the present study. The global food market for HPP is currently estimated at ca. USD $14 billion, and it is anticipated to reach ca. USD $27 billion in 2023 and then rise to ca. USD $50 billion by 2027 [35]. The projected growth of HPP is fueled, to a large extent, by an ever-increasing demand from consumers for clean label, high convenience, low preservative, and minimally processed foods [36]. Our data substantiate that HPP is an effective alternative to heat for inactivating STEC in raw meatballs and it does not result in appreciable untoward effects on product quality. Subjecting inoculated meatballs to pressures of 400 or 600 MPa for up to 18 min was sufficient to deliver reductions of 0.9 to 3.0 log CFU/g in STEC levels. This is significant as STEC levels typically would be low in raw ground beef and ground veal, and as additional lethality will be achieved by subsequent cooking. Lowering the recovery rate and pathogen levels in (raw) meatballs by HPP will presumably also lower the public health risk from STEC.

## Figures and Tables

**Table 1:** D-values (min) for Shiga toxin–producing *Escherichia coli* in refrigerated and frozen meatballs pressure treated at 400 or 600 MPa (N = 3 trials, *n* = 3 samples per trial).

State of Meat	Pressure (MPa)	Type of Meat		
		Veal	Beef	Mixture ^1^
Refrigerated	400	7.10	6.54	6.84
	600	1.14	1.45	1.32
Frozen	400	5.69	5.89	5.90
	600	3.86	3.60	3.56

^1^ Mixture of ground beef, pork, and veal.
